# Hospital Notes

**Published:** 1871-09

**Authors:** C. C. F. Gay

**Affiliations:** Surgeon to the Buffalo General Hospital


ART. III.—*Hospital Notes*, by C. C. F. GAY, M.D., Surgeon to the Buffalo General Hospital.

*Refracture of right tibea and fibula at their lower third.*

Thomas Oates, of Michigan, aged 35 years, fractured his tibia and fibula on June 2nd, 1870. He entered Hospital May 20th, 1871. There is firm ossific union, the upper fragment overlaps the lower and forms an ugly prominence as well as depression. The limb is bent considerably from a straight line, is two and one-half inches shortened, the ankle joint is anchylosed, the tendo Achillis is contracted, and he has not been able to walk at all, or step with the limb. He manages to get about upon crutches.

Chloroform was given the patient on May 27th, 1871, when I succeeded in refracturing the leg, breaking up completely the union at the original point of fracture, bending the leg in any direction, and then dividing subcutaneously the tendo-achilis. The patient was now put to bed and extention, and counter-extention used. Extension was applied from a shoe constructed with a wooden sole. The leather portion of the shoe extended up, and was laced around the ancle. On July 31st, I have noted, that "the limb is greatly improved." The condition of the limb August 14th is such as to justifiy this operation for refracture. The leg is no longer,* the muscles were so rigid that extention accomplished nothing; but the leg is now straight, with the deformity much diminished, and the patient is able to do some walking about the wards without the aid of crutch or cane.

The refracture of a bone is an expedient of doubtful propriety, and I believe the Surgeon would scarcely be justified in resorting to it unless there was great deformity, and almost entire inability to use the limb, and a desire expressed also on the part of the patient that refracture should be resorted to; and then I think the Surgeon would not be justified if his object was only to lengthen the limb, since he would surely be doomed to disappointment, inasmuch as the muscles, after fracture of considerable duration, appear to have become shortened as well as rigid, and no amount of power applied will be sufficient to overcome the contraction and rigidity of them. But, if the intention is to more accurately adapt the fractured extremities and to relieve deformity, when also there might be reasonable expectations that the limb could be extended, then, the Surgeon, I believe, is justifiable in resorting to this doubtful expedient, and of subjecting his patient to the painful ordeal.

*Necrosis of the Cranial Bones Trephining—Recovery.*—Mr. B. German, aged 41 years, of good physique, no constitutional vice, has never had syphilis. Two years since patient received direct blow upon the head, since which there has been pain, swelling, and more or less discharge of pus. The pupils of the eyes are largely dilated, and he thinks he has had slight convulsions. On exploration of the wound with the finger I find the bone roughened, necrosed, and a portion exfoliated at the posterior superior angles of the parietal

* September 13. Patient says his leg is lengthened one inch.

bones. On May 22nd, 1871, the patient was given chloroform, the flap dissected up and the bones found necrosed over a space measuring two and a-half inches in diameter. All the diseased portion of bone was removed, leaving an open space exposing the meninges of the brain two and a-half inches in diameter. In removing the greater portion of the bone the gnawing forceps were made use of. The flap was replaced and secured by sutures. This patient, with several others in the Hospital, was attacked with erysipelas, from which he recovered, as well as from the disease for which the operation was performed, leaving Hospital about June 10th, with wound closed, and with no more supuration. There have been no convulsions since the operation, and the pupils of the eyes have resumed their normal size.

*Trachiotomy*—Child, two and a-half years of age, had membraneous croup, was sick three days, respiration very difficult, and at times performed mechanically; face livid, pulse feeble and rapid. The child was certain to die unless Trachiotomy could relieve, and this was the only remaining hope that could be held up as in any way adequate to saving the life of the little sufferer, and this was advised. Assisted by Drs. Harrington and Parker, I performed the operation, which gave almost instantaneous relief to the patient. There was escape, on introducing the tube, of considerable false membrane, respiration became normal, the face returned to its usual color, the pulse was diminished in frequency, and the child able to sit and stand up; and for a time the case looked promising for ultimate recovery. The trachial tube would fill up with thick tenacious mucus so firmly adhering to the sides of the tube as to necessitate cleaning every half hour.

The child lived thirty-six hours after the operation, during all the time relieved from the distress attendant upon the disease prior to operation. *Post mortem* examination showed the trachial tube above the insertion of the tube most completely corked up with false membrane, old and recent.

The final termination of this case furnishes no argument against the propriety of making the operation; but on the contrary, furnishes an argument in favor of the operation. It is a part of the duty and province of the Surgeon and Physician to relieve suffering, if he cannot cure disease. The pain inflicted by an operation, if the patient be well under the influence of chloroform, is *nil*, while the comfort of relief to both patient, and parents, and friends, afforded by the prolongation of a human life, and the subsequent exemption from suffering would seem to be good and sufficient reasons why the operation should be made in all cases.

